# Drivers of prohibited natural resource collection in Chitwan National Park, Nepal

**DOI:** 10.1017/s0376892922000121

**Published:** 2022-04-07

**Authors:** Scott T Yabiku, Abigail Sullivan, Abigail M York, Qunshan Zhao, Jennifer E Glick, Sharon J Hall, Dirgha J Ghimire, Li An

**Affiliations:** 1 Penn State University, 306 Oswald Tower, Penn State University, University Park, PA 16802, USA; 2 Boston University Earth & Environment, 685 Commonwealth Avenue, Boston, MA 02215, USA; 3 School of Human Evolution and Social Change, Arizona State University, Tempe, AZ 85287, USA; 4 Urban Big Data Centre 7-302, 7 Lilybank Gardens, University of Glasgow, Glasgow, G12 8RZ, UK; 5 Penn State University, 601 Oswald Tower, Penn State University, University Park, PA 16802, USA; 6 School of Life Sciences, Arizona State University, Tempe, AZ 85287, USA; 7 Population Studies Center, University of Michigan, 426 Thompson St, Ann Arbor, MI 48106, USA; 8 San Diego State University, 5500 Campanile Drive, San Diego, CA 92182-4493, USA

**Keywords:** community forestry, illegal collection, invasive species, Nepal, prohibited behaviours, protected areas, social-ecological system

## Abstract

Protected areas (PAs) are critical for achieving conservation, economic and development goals, but the factors that lead households to engage in prohibited resource collection in PAs are not well understood. We examine collection behaviours in community forests and the protected Chitwan National Park in Chitwan, Nepal. Our approach incorporates household and ecological data, including structured interviews, spatially explicit data on collection behaviours measured with computer tablets and a systematic field survey of invasive species. We pair our data with a framework that considers factors related to a household’s demand for resources, barriers to prohibited resource collection, barriers to legal resource collection and alternatives to resource collection. The analysis identifies key drivers of prohibited collection, including sociodemographic variables and perceptions of an invasive plant (*Mikania micrantha*). The social-ecological systems approach reveals that household perceptions of the presence of *M. micrantha* were more strongly associated with resource collection decisions than the actual ecologically measured presence of the plant. We explore the policy implications of our findings for PAs and propose that employing a social-ecological systems approach leads to conservation policy and scientific insights that are not possible to achieve with social or ecological approaches alone.

## Introduction

Protected areas (PAs) have been set aside for specific management goals (Geldmann et al. 2019). Globally, the total area designated protected has increased dramatically, from negligible in the early twentieth century to *c.* 22.5 million km^2^ of land and 28.1 million km^2^ of sea in 2021, although PAs are often chosen based more on political considerations than scientific reasoning (Watson et al. 2014, UNEP-WCMC & IUCN 2021). PAs now strive to balance social, natural and economic goals (Gutiérrez et al. 2011). Although the success of many PAs has been lauded, some PAs have been considered failures (Wilkie et al. 2001, Edgar et al. 2014). These failures are frequently driven by inattention to the sociopolitical context in the initial design stages (Chuenpagdee et al. 2013, Watson et al. 2016). One important gap in understanding why PAs fail is unpacking the motivations of people who violate rules meant to protect these areas.

There are challenges to studying why some people abide by rules within PAs while others disregard them. First, it is difficult to obtain direct reports on rule violations from violators because violations are often either legally prohibited or socially unacceptable. Prior work has typically relied on indirect measures, although some studies have engaged in direct questioning or observation of violators (e.g., Yonariza & Webb 2007, Bergseth et al. 2017). Second, even if rule violations are measured, information on the violations are rarely spatially explicit (Hofer et al. 2000, Knapp et al. 2017). Without understanding the spatial distribution of rule violations, it is difficult to maximize the efficiency of enforcement resources, including the distribution and intensity of guard or patrol efforts (Hofer et al. 2000). Third, many studies of rule violators often lack representative samples of the human populations that use local resources (e.g., Mann 1995, Ward et al. 2018). While non-representative studies contribute unique and rich descriptions, they may incompletely describe prohibited collection behaviours.

PAs are complex social-ecological systems in which multiple pathways and mechanisms affect each other (Cumming & Allen 2017, Picone et al. 2020). Due to disciplinary separation, social and ecological processes were in the past often studied separately or with minimum consideration of their interaction with one another. With human–environment systems (Turner et al. 2003) or coupled human and natural systems (Liu et al. 2007), the social-ecological systems (SESs) framework assumes that such systems are integrated and complex, with humans and natural subsystems interacting with one another (Dietz et al. 2003). Studies of prohibited natural resource collection typically focus on a single part of the system and on data relating to ranger patrol observations (Critchlow et al. 2015), respondent self-reports (Nuno et al. 2013), spatial mapping (Faulkner et al. 2018) or stakeholder perspectives (Kahler et al. 2013). We argue that multiple data perspectives are needed to better understand prohibited collection in complex social-ecological systems. Note that many studies use the term ‘illegal’ when describing prohibited natural resource collection activities, but we specifically use ‘prohibited’ as opposed to ‘illegal’ due to the negative and inappropriate implication that community members collecting in PAs are criminals.

The objectives of this research were to: (1) collect human data on the household drivers of prohibited collection and perceptions of *Mikania micrantha* in community forests; (2) collect institutional data from community forests managers on the perceived spatial distribution of *M. micrantha*; (3) collect ecological data on the spatial distribution of *M. micrantha*; and (4) examine the impacts of these human, institutional and ecological factors on prohibited collection. Each of our data sources benefits from: (1) direct measurement, (2) spatial precision and (3) representative sampling. We focus on *M. micrantha* because it has been called the ‘most serious weed in the tropical and subtropical areas of Nepal’ (Baral & Adhikari 2017), and our fieldwork with residents, forest managers and non-governmental organization (NGO) officials indicated it to be the most concerning invasive species in the region. Although our setting is the area surrounding Chitwan National Park (CNP) in Nepal and the prohibited resources are fuelwood and fodder within park boundaries, we believe our comprehensive approach to prohibited collection is applicable to many complex social-ecological systems.

## Methods

### Research setting

The western Chitwan Valley of Nepal lies at 120–815 m elevation in the subtropical Terai region of Nepal, characterized by elongated valleys created by faults within the foothills of the actively uplifting Himalaya plateau (Lehmkuhl 1994). Much of the area was deforested and settled in the 1950s and 1960s, and today the 250-km^2^ western Chitwan Valley is home to over 200 000 individuals (Central Bureau of Statistics – Nepal 2016). While the northern region is intensively cultivated, the southern and western regions are dominated by CNP, a 932-km^2^ UNESCO World Heritage Site established in 1973 and internationally recognized for its largely intact primary forests, grasslands and habitats for endangered and vulnerable, charismatic mammal species. CNP is home to the vulnerable one-horned rhinoceros (*Rhinoceros unicornis*) and endangered Bengal tiger (*Panthera tigris tigris*) (Spiteri & Nepal 2008). The Forest Act 1993 created the existing community forest (CF) system – a type of decentralized, locally governed forest management – to prevent habitat degradation and to sustain the quality of life for growing numbers of buffer zone (the area surrounding CNP) residents (Nagendra 2002). This arrangement allows for residents to legally collect resources from CF areas while CNP is rendered off-limits (Jones 2007).

Areas near CFs and CNP are experiencing rapid human population growth and are threatened by three of the world’s 100 worst invasive exotic species: *M. micrantha*, *Chromolaena odorata* and *Lantana camara* (ISSG 2000). *M. micrantha* is particularly problematic, spreading quickly and degrading resources in these important landscapes (some CFs have lost up to 70% of their forest area to *M. micrantha*) (Clark 2020). *M. micrantha*, or ‘mile-a-minute weed’, was discovered in the Chitwan region in the early 1990s and is thought to have spread during a large flooding event (Paudyal 2007). *M. micrantha* is fire-adapted (Swamy & Ramakrishnan 1988), and it is thought to proliferate in high-nutrient, high-light and high-moisture conditions (Swamy & Ramakrishnan 1988, Zhang et al. 2004). Together, human population growth and increasing numbers of invasive species may lead to overburdened CFs and increase the appeal of prohibited collection in CNP.

### Data collection

To understand prohibited collection, we collected data across multiple domains: human, ecological and institutional. In each domain, we ensured that our data were direct, spatially explicit and representative. Human subjects research was approved by the Institutional Review Board at Penn State University (Study #00005177).

#### Human data.

Household data were collected in autumn 2014. Social surveys included questions about the household, including structure, ethnicity, number of animals, area of land farmed, whether the household buys fodder and fuelwood and experiences with invasive species. To assess the spatial aspect of collection behaviours, a knowledgeable household member used an interactive tablet app that contained touch-enabled satellite imagery of the Chitwan area. The respondent circled areas where household members had collected different types of forest resources in the past year. Using tablets to collect spatial data in this population has been shown to be accurate (Yabiku et al. 2017). The tablet app did not show CF or park boundaries (which could have influenced willingness to indicate collection in the CNP), and we did not ask about a prohibited activity (e.g., ‘Does your household collect fuelwood in the park?’), which could raise social desirability bias (Krumpal 2013). In addition, using the tablet app provides greater spatial accuracy than responses to survey questions (Yabiku et al. 2017).

**Fig. 1.**
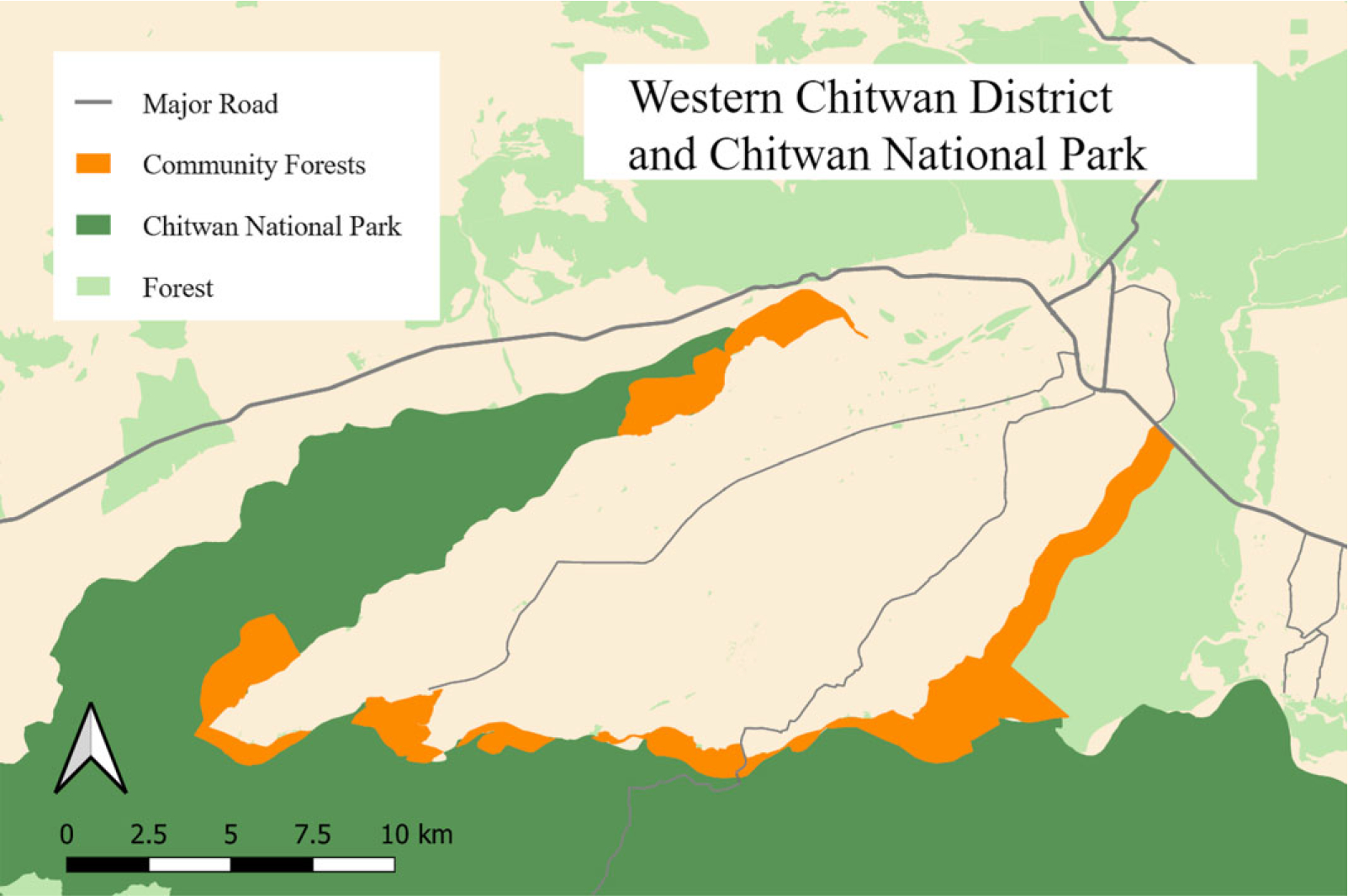
Western Chitwan District and Chitwan National Park. *Sources:* Open Street Map, Department of National Parks and Wildlife Conservation, Ministry of Forest, Nepal, and primary data collection.

Although the household survey interviewed 1235 households, our analysis uses 1036 households that resided in areas eligible for membership in one of 21 local CFs; we refer to these areas as ‘CF catchment areas’. Of the 21 CFs, 17 are within buffer zone CFs (BZCF) under the jurisdiction of CNP (Department of National Parks and Wildlife Conservation). The remaining four CFs are under the jurisdiction of the District Forest Office (Department of Forest). Although all share governing principles, the BZCFs receive additional financial resources from CNP for forest management, compensation for damage by wildlife and community development activities. Figure 1 shows the CF areas in relation to their proximity to CNP.

Households were selected through a three-stage process: first, wards (administrative units) in catchment areas were randomly sampled proportionate to population size in the 2011 Census; second, chosen wards were divided into equal sub-wards of *c.* 400 households each and sub-wards were randomly sampled; third, chosen sub-wards were enumerated to create a household sampling frame and households were randomly sampled. The household response rate for the face-to-face survey interview was 98%.

### Ecological data

The ecological data were collected from 2013 to 2015 using a systematic vegetation survey throughout the CFs that surround the household settlements: 11 forests were surveyed in 2013 (August–November), 8 in 2014 (September–November) and 2 in 2015 (September–October). In the CFs, parallel transects were drawn every 200 m. Along each transect, one plot (composed of two 5 m × 5 m areas) was sampled every 50 m. Variables collected included canopy cover, evidence of disturbance, presence as well as percentage cover of the invasive species *M. micrantha*, *C. odorata* and *L. camara* and percentage cover of the three most dominant herbs and tree species. In total, 2219 plots were surveyed.

### Institutional data

We measured both self-governance and market institutions. A representative from each of the 21 CF management committees was interviewed in 2014. Surveys measured management techniques, perceived challenges and experiences with invasive species. In addition, the representatives used a tablet to indicate areas of invasive species presence in their CF. Market organizations, governmental organizations and NGOs were enumerated in terms of the presence of organizations such as shops, schools, health clinics, restaurants and factories. Project staff walked all roads in each sub-ward and used a custom-designed tablet app with integrated GPS to enumerate the locations of all organizations. In the 21 catchment areas, 2335 such organizations were identified.

### Data analysis

#### Dependent variable

Prohibited collection in CNP over the past year was directly collected from a household respondent. If the respondent drew a collection area for fuelwood or fodder (the most commonly collected resources) that overlapped with CNP boundaries, the household was coded 1 on prohibited collection and 0 otherwise.

#### Covariates of prohibited collection

We expected that the likelihood that a household engaged in prohibited collection would vary according to the following factors derived from our observations and existing literature (Shova & Hubacek 2011): (1) demand for resources; (2) barriers to prohibited resource collection; (3) barriers to legal resource collection; and (4) alternatives to resource collection.

The demand for forest resources (fodder and firewood) was hypothesized to be positively associated with prohibited collection. Variables representing the demand for forest resources included the number of household members, number of dairy animals and size of farmland. Barriers to prohibited resource collection were hypothesized to reduce prohibited collection. This variable was measured according to the distance from the household to the nearest CNP boundary; greater distance made collection in CNP less convenient. Barriers to legal resource collection were expected to increase prohibited collection. Barriers included minority ethnicity (who are sometimes discouraged or excluded from CF membership), being in a catchment area with a small CF and being a member of a CF with a higher presence of the invasive species *M. micrantha*. Reflecting the multiple dimensions through which invasive species may increase prohibited collection, we measured *M. micrantha* in three ways: household perceptions of *M. micrantha* spread; the percentage of the CF covered by *M. micrantha* as indicated in the management committee survey; and the percentage of a CF’s plots with any *M. micrantha* (i.e., of all surveyed plots in a CF, we calculated the percentage of surveyed plots in each CF with any *M. micrantha* presence). Alternatives to resource collection were hypothesized to lower the likelihood of prohibited collection. Households with more alternatives were those with greater incomes, that report purchases (in addition to or instead of collection) of fuelwood and animal feed and that are in areas with a high density of market (non-governmental) organizations where alternatives to resource collection were readily available. Table 1 shows the data sources of the variables used in the analysis and provides details of how they were coded. Although we did not include the household members’ occupations, many households retained some agricultural activity even when a member had a non-farm occupation. Thus, even without an occupation variable, we believe a household’s dependency on natural resources was captured by the more proximate indicators (e.g., land area, dairy animals).

Although ethnic groups are diverse, for analysis we adopted five categories from the literature (Axinn & Yabiku 2001): (1) Brahmin/Chhetri (historically the most advantaged); (2) Hill Janajati (moved to Chitwan from the hill regions; Tibeto-Burmese origin groups includes Tamang, Gurung, Magar, Rai and others, as well as Gurkhas); (3) Dalit (historically the most disadvantaged); (4) Newar (historically merchants and engaged in commerce and commercial business); and (5) Terai Janajati (indigenous to Nepal’s plain areas and include Tharu, Kumal, Bote and others).

#### Statistical modelling

Because our dependent variable is dichotomous, we used logistic regression to predict whether a household engaged in prohibited resource collection. To avoid skew, we applied a log transformation to several continuous predictors that had large ranges: park distance, CF size (area) and density of market (non-governmental) organizations. The sampling of households was clustered by CF catchment area. To accommodate for this, we included a random intercept for catchment area in our model. In random-effects logistic regression models, the coefficient can be interpreted as the relationship between the independent variable and the log odds of prohibited resource collection. A positive coefficient means a variable increases the log odds of prohibited collection; a negative coefficient decreases the log odds of prohibited collection. The models were estimated in *R* version 4.0.4 (R Core Team 2021) using the package *lme4* version 1.1–26 (Bates et al. 2015).

## Results

Of primary interest is the dependent variable: the percentage of households that reported prohibited resource collection in CNP, which is *c.* 11% (Table 2). This small percentage suggests that collection in CNP was an uncommon behaviour, in contrast to a behaviour that might be against the rules but was frequently performed by many community members.

There was diversity in the factors that could increase or decrease a household’s tendency to collect resources in CNP (Table 2). Some households had substantial farm holdings (up to 88 *kattha*; *c.* 3 ha), while others had no land. On average, households were *c.* 2.25 km from the closest CNP boundary, although this distance varied from 50 m to nearly 10 km. Perceived *M. micrantha* invasion in a household’s assigned CF (based on the catchment area) varied across households. Approximately 20% of households thought *M. micrantha* was decreasing or not changing and *c.* 20% thought it was increasing slowly, but the largest group, at 50%, thought it was increasing rapidly; *c.* 10% answered ‘did not know’ to this question. When asked to circle the areas in which they believed *M. micrantha* was present, the households’ CF managers indicated areas that, on average, it encompassed 47% of their forest. As measured in our 2219 ecological plot surveys, *M. micrantha* was present, on average, in 14% of plots in the CF of which a household was a member. The managers and plot survey reports differed greatly.

We present our model with all predictors (Table 3), but we also tested predictors in sets so as to determine whether the results in our final model could have been affected by collinearity between predictors; the results were no different than if sets of predictors were estimated separately.

The amount of land farmed, number of dairy animals (cows, buffaloes, sheep and goats) owned and household size were not associated with the log odds of prohibited collection (Table 3). The logged distance from the respondent household to CNP was significantly associated with prohibited resource collection in the hypothesized direction. In other words, household distance from CNP represented a barrier to prohibited resource collection and so the log odds of prohibited resource collection were lower.

With respect to barriers to legal collection in the CFs, household perception of *M. micrantha* invasion in a CF was significantly associated with the log odds of prohibited collection. Compared to households that said *M. micrantha* was decreasing or staying the same (the reference group), households that thought *M. micrantha* was rapidly increasing were significantly more likely to engage in prohibited resource collection. The frequency of *M. micrantha* in plots in the household’s assigned CF, as measured in our ecological surveys or in the CF management committee tablet surveys, was not significantly associated with prohibited collection (Table 3). The logged area of the household’s CF was not associated with prohibited resource collection. Regarding alternatives to forest resources, households that bought more fodder and firewood were significantly less likely to engage in prohibited collection, but the density of market organizations and NGOs was not related to prohibited collection. The caste of the household, which was included as a control variable, was significantly associated with collection in CNP. Compared to Brahmin (the reference group), Hill Janajati, Dalit and Terai Janajati demonstrated significantly greater log odds of prohibited collection.

## Discussion

### A systems approach to studying prohibited natural resource collection

We detailed a social-ecological systems approach, analysing prohibited natural resource collection in CNP by integrating social, institutional and ecological data. This approach gave us a holistic understanding of prohibited natural resource collection, and we believe incorporating social, institutional and ecological data provides unique insights into the drivers of prohibited collection behaviours. An understanding of the system, including its social and ecological characteristics, may be necessary to inform successful management policies for PAs (Carter et al. 2017).

### Drivers of prohibited natural resource collection in Chitwan National Park

None of the measures that we expected to increase the demand for forest resources were associated with prohibited collection, but measures of other factors were predictive in expected ways. Households that perceived *M. micrantha* as rapidly increasing and those that belonged to traditionally disadvantaged and marginalized ethnic groups were more likely to participate in prohibited resource collection. In addition, households that did not buy alternatives to resource collection and those closer to CNP also were more likely to take part in prohibited collection.

There are several potential explanations for the finding that a household’s perception of *M. micrantha* invasion was a predictor of prohibited collection, but *M. micrantha* invasion measured from other perspectives (our representative ecological surveys and the community forest committee tablet assessment) did not predict prohibited collection. Human behaviour is often motivated by perceptions and attitudes (Schlüter et al. 2017), and it is possible that perceptions of *M. micrantha* invasion that are misaligned with ecological measurements of *M. micrantha* invasion are influential in prohibited resource collection. In addition, people’s perceptions may be incomplete. Distrust between CF members, CF officials and CNP officials has been a barrier to gaining information regarding *M. micrantha* (Sullivan et al. 2017). Furthermore, our ecological surveys measured entire CFs using systematic sampling. The areas of the CFs that households most frequently visit are often only a fraction of the entire CF. Similarly, CF officials’ perceptions of *M. micrantha* probably also were influenced by selective observation.

Our finding that households from traditionally marginalized ethnic groups are more likely to participate in prohibited collection in CNP is consistent with historical context, as Nepal’s government has a history of formally and informally marginalizing non-Brahmin ethnicities (Nightingale 2011). Much of this exclusion results from injustices against Indigenous peoples and their dispossession from their native lands (Maharjan 2017). PAs have been established in Nepal without the consent of the Indigenous peoples who have historically lived there (Stevens 2013). In some cases, Indigenous groups have continued to reside in PAs in protest and have established their own Indigenous Peoples and Community Conserved Territories and Areas (e.g., within Nepal’s four high Himalayan national parks) (Stevens 2013). In CNP and other Terai and western national parks, Indigenous peoples including the Tharu people were displaced as PAs were established or expanded (Basnet 2017). The consequences of this injustice persist. Households belonging to native ethnic groups often have higher poverty, fewer material resources and fewer formal social support structures, such as equal access to the legal collection of CF resources (Sullivan et al. 2016).

### Crafting successful policies to deter resource collection in protected areas

Policies need to carefully define and understand what poverty and marginalization mean in a given situation and link these to drivers of prohibited hunting, poaching or resource collection (Duffy et al. 2016). Many conservation studies rely on economic definitions that emphasize income measures and ignore structural context, including cultural and ethnic relationships. Prohibited wildlife hunting and natural resource collection may be tied to prestige, customs and local cultural beliefs, going beyond reasons such as a lack of access (Montgomery 2020). Beyond providing paid labour and other market-based remedies, the results of this study point to the need to measure multidimensional aspects of poverty and marginalization. Ultimately, understanding the local context (e.g., histories of discrimination) in relation to engaging in prohibited natural resource collection is an important policy foundation.

In Chitwan, some qualitative work and our quantitative models indicate that collecting prohibited resources from CNP may be related to a lack of adequate natural resources being obtainable via legal means (e.g., within the CF or through purchase). Conservation policies that address unequal legal access to natural resources may yield beneficial outcomes. Differences between specific policies may be less important to the success of the policy than having policies that are linked to local intrinsic motivations and social justice issues (Cetas & Yasué 2017). A conservation policy in Chitwan that addresses local motivations for resource collection requires an understanding that access to CF resources can be unequal across multiple dimensions.

Conservation policies often displace social-environmental pressures to surrounding lands, which may not be legally protected by the formal policies of adjacent PAs (Dou et al. 2018). These spillover effects complicate efforts to create conservation policies that address structural inequalities in natural resource access and livelihoods (Ma et al. 2020). In Chitwan, the formal rules of the community forestry programme have helped to conserve the forest land governed by those rules (Thing & Poudel 2017), but it is possible that those same rules have shifted resource collection to adjacent forests that are not governed by community forestry policy. To avoid the unintended consequence of degrading the land surrounding PAs, conservation policies must be holistic (Dou et al. 2018). Potential spill-over effects in Chitwan are connected to underlying issues of unequal access to natural resources. Policies that can address these effects will need to confront this inequality in order to be successful.

One possibility for addressing inequality in access to forest resources in Chitwan is creating a programme to build local capacity – in terms of both logistical resources and shifting social norms – so as to redistribute resources between neighbouring CFs to provide for low-income households, for recently established households and for those facing other hardships. Our social-ecological systems approach also revealed that it is critical for managers and stakeholders to consider both social and ecological data in order to fully understand people’s resource collection decisions. This is important because household perceptions of *M. micrantha* as rapidly increasing did not always align with the ecological data on *M. micrantha*. Local officials with broader knowledge of *M. micrantha* may be able to guide households in collecting forest resources in areas with less *M. micrantha*, but only if households have established trust with these officials (Shrestha et al. 2019). Thus, strengthening relationships between local conservation officials (e.g., CNP and conservation NGO officials) and local households should be included as part of any policy to reduce prohibited resource collection in CNP. Policies that address the drivers of prohibited natural resource collection, such as supporting traditionally marginalized households and addressing perceptions of *M. micrantha*, are critical to reducing prohibited natural resource collection in PAs (Roe 2015). Community members should be involved in the development of any programme designed to reduce prohibited resource collection (Nyaupane et al. 2020) in order to increase the likelihood that social, political and other structural factors will be addressed.

Individual conservation programmes are not capable of overturning or reversing structural issues such as multidimensional poverty and historical mistrust between officials in power that often drive prohibited natural resource collection (Duffy et al. 2016). Any policy to reduce prohibited natural resource collection in CNP, and indeed any PA, must attempt to address these underlying issues as opposed to solely targeting individual behaviours. This is why, based on our results, we propose establishing policies that build and strengthen the community-level capacity to elevate and support households that lack access to necessary natural resources. In our case, the underlying assumption is that households are engaging in prohibited resource collection in CNP because they need access to unequally distributed resources or resources that they perceive as having been invaded by *M. micrantha*. Thus, supporting relationship-strengthening among households and conservation officials and capacity-building for equitable resource access should reduce prohibited collection.

## Figures and Tables

**Table 1.** Variables and data sources used in the analysis of prohibited resource collection in Chitwan National Park (CNP), Nepal.

Variable	Data source	Details

Household prohibited resource collection	Tablet data from household survey	Households that indicated firewood or fodder resource collection within CNP were coded 1, 0 otherwise
*Demand for forest resources*		
Household member count	Household survey	Number of people living in the household
Household dairy animals count	Household survey	Number of dairy animals owned by the household
Household farmland size (*kattha*)	Household survey	Size of land farmed by the household
*Barriers to prohibited resource collection*		
Distance from household to park	GPS from household survey, CNP shapefiles	Distance in km from household to the nearest CNP boundary
*Barriers to legal resource collection*		
Size of household’s CF	CF shapefiles	Area in km^2^
Household’s perceived *Mikania micrantha* spread in forest	Household survey	Respondent indicated whether they thought *M. micrantha* in the forests was decreasing, not changing, increasing slowly, increasing rapidly or did not know
CF managers’ reported *M. micrantha* percentage coverage in forest	Tablet data from CF manager survey	Managers indicated areas in the forests where they believed *M. micrantha* was present
Measured proportion of forest plot surveys with *M. micrantha* present	Ecological plot survey	Average presence of *M. micrantha* across the surveyed plots within each CF was calculated; this value was matched to each household assigned to the respective CF
*Alternatives to resource collection*		
Scale at which household bought firewood and fodder	Household survey	Households indicated whether they purchased firewood and/or fodder; each type of purchase added 1 to the scale
Density of nearby market/non-governmental organizations	Organizational survey	The density of market/non-governmental organizations in each household’s neighbourhood
*Controls*		
Caste	Household survey	Main caste and ethnic groups in Chitwan (Brahmin, Hill Janajati, Dalit, Newar and Terai Janajati) coded into dummy variables
Household income	Household survey	Coded 1 if household reported and income of >50 000 Nepalese rupees, 0 otherwise

CF = community forest.

**Table 2.** Descriptive statistics of households (n = 1036) in Chitwan National Park (CNP), Nepal. Percentages are given for categorical variables; means and standard deviations are given for continuous variables.

Variable	Percentage	Mean	SD

Household reported prohibited resource collection (dependent variable)	10.7		
*Demand for forest resources*			
Household member count		5.27	2.24
Household dairy animal count		3.08	3.16
Household farmland size (*kattha*)		10.0	9.95
*Barriers to prohibited resource collection*			
Distance from household to nearest CNP boundary (km)		2.25	2.15
*Barriers to legal resource collection*			
Size of household’s community forest (km^2^)		1.90	1.74
Household’s perceived *Mikania micrantha* spread in forest: decreasing or not changing	19.4		
Household’s perceived *M. micrantha* spread in forest: gradually increasing	19.9		
Household’s perceived *M. micrantha* spread in forest: rapidly increasing	50.2		
Household’s perceived *M. micrantha* spread in forest: do not know	10.5		
Community forest managers’ reported *M. micrantha* percentage coverage in forests		0.47	0.29
Measured proportion of forest plot surveys with *M. micrantha* present		0.14	0.22
*Alternatives to resource collection*			
Scale at which household purchased firewood and fodder		0.81	0.74
Density of nearby market/non-governmental organizations per km^2^		30.84	2.80
*Controls*			
Caste: Brahmin	50.5		
Caste: Hill Janajati	16.0		
Caste: Dalit	12.7		
Caste: Newar	3.7		
Caste: Terai Janajati	17.1		
Household income >50 000 Nepalese rupees	68.9		

**Table 3.** Results of a random intercept logistic regression model (n = 1036) of variables associated with household prohibited resource collection in Chitwan National Park, Nepal.

Variable	Estimate	SE	p

*Demand for forest resources*			
Household member count	0.05	0.05	0.35
Household dairy animals count	0.02	0.04	0.74
Household farmland size (*kattha*)	0.01	0.01	0.70
*Barriers to prohibited resource collection*			
Distance from household to nearest park boundary, logged	−0.57^a^	0.24	0.02
*Barriers to legal resource collection*			
Size of household’s community forest, logged	−0.42	0.31	0.17
Household’s perceived *Mikania micrantha* spread in forest: gradually increasing^b^	−0.02	0.44	0.96
Household’s perceived *M. micrantha* spread in forest: rapidly increasing^b^	0.76^a^	0.34	0.03
Household’s perceived *M. micrantha* spread in forest: do not know^b^	−0.40	0.62	0.52
Community forest managers’ reported *M. micrantha* coverage in forests	−0.05	1.41	0.97
Measured proportion of forest plot surveys with *M. micrantha* present	0.09	1.67	0.96
*Alternatives to resource collection*			
Scale at which household purchased firewood and fodder	−0.58^a^	0.19	<0.01
Density of nearby market/non-governmental organizations, logged	0.33	0.28	0.24
*Controls*			
Caste: Hill Janajati^c^	1.05^a^	0.39	0.01
Caste: Dalit^c^	1.16^a^	0.39	<0.01
Caste: Newar^c^	0.88	0.65	0.18
Caste: Terai Janajati^c^	1.40^a^	0.40	<0.01
Household income >50 000 Nepalese rupees	−0.33	0.25	0.19
Intercept	−4.48^a^	1.19	<0.01

a 95% confidence interval excludes zero.

b Reference group for dummy variables is ‘Household perceived *M. micrantha* is decreasing or not changing’.

c Reference group for dummy variables is ‘Caste: Brahmin’.
